# Patient Health Record Smart Network Challenges and Trends for a Smarter World

**DOI:** 10.3390/s25123710

**Published:** 2025-06-13

**Authors:** Dragoş Vicoveanu, Ovidiu Gherman, Iuliana Șoldănescu, Alexandru Lavric

**Affiliations:** 1 Faculty of Electrical Engineering and Computer Science, Ștefan cel Mare University of Suceava, 720229 Suceava, Romania; dragos.vicoveanu@usm.ro (D.V.);; 2 Integrated Center for Research, Development and Innovation for Advanced Materials, Nanotechnologies, Manufacturing and Control Distributed Systems (MANSiD), Stefan cel Mare University of Suceava, 720229 Suceava, Romania

**Keywords:** patient health records, Internet of Things, artificial intelligence, blockchain, PHR architectures and systems

## Abstract

Personal health records (PHRs) are digital repositories that allow individuals to access, manage, and share their health information. By enabling patients to track their health over time and communicate effectively with healthcare providers, personal health records support more personalized care and improve the quality of healthcare. Their integration with emerging technologies, such as the Internet of Things (IoT), artificial intelligence (AI), and blockchain, enhances the utility and security of health data management, facilitating continuous health monitoring, automated decision support, and secure, decentralized data exchange. Despite their potential, PHR systems face significant challenges, including privacy concerns, security issues, and digital accessibility problems. This paper discusses the fundamental concepts, requirements, system architectures, and data sources that underpin modern PHR implementations, highlighting how they enable continuous health monitoring through the integration of wearable sensors; mobile health applications; and IoT-enabled medical devices that collect, process, and transmit data to support proactive care and personalized treatments. The benefits and limitations of PHR systems are also discussed in detail, with a focus on interoperability, adoption drivers, and the role of advanced technologies in supporting the development of secure and scalable health information systems for a smarter world.

## 1. Introduction

In recent decades, advances in health information technology have transformed the healthcare industry worldwide, introducing new concepts in healthcare applications and services that integrate medical information into technology models. In addition, the growing number of patients generating large amounts of data, from genomics to the Internet of Things (IoT) connecting individuals to devices, has created new challenges [1].

The development of mobile computing and the proliferation of wearable sensor devices have exponentially increased the number of health records to be managed [2,3,4]. On the other hand, healthcare organizations face real challenges in organizing this vast amount of medical data, especially when considering the correlation among healthcare stakeholders. This applies to all processes that implement the collection, exchange, and management of health information for individuals and populations [5,6].

In addition, the medical systems face difficulties in communicating with elderly patients or patients with various dysfunctions, such as those with neurodegenerative diseases, which hinders the outcomes of treatments and leads to medical errors. Not only does communication with the patient affect the diagnosis, but miscommunication between the medical staff can also cause errors in the diagnostic process [7]. The use of personal health records could help significantly reduce these errors and make the work of physicians easier. Especially in the case of chronically ill patients, close monitoring is essential to control disease progression, manage treatments, or follow up medical consultations to ensure that the patient is receiving appropriate care [8].

The scientific literature distinguishes three types of patient health records (PHRs): stand-alone PHRs, where the patient enters personal health information that is managed and maintained entirely by the patient, independent of healthcare providers or institutions (i.e., a patient logs of medications, allergies, or diagnoses, in a Notepad or MS Excel file, etc.); institution-specific PHRs that are accessible to the institution’s medical staff but also to the patient through a web portal, and finally, integrated/networked PHRs that can connect and share information at the macro level [7]. The global adoption of PHRs depends on many factors, including digital infrastructure, patient education, medical staff training, technological advances, and the commitment of healthcare providers. A small survey of approximately 830 people, who were asked about the use of PHRs, found that 83% found the use of PHRs to be useful. And of the 62 people who have access to PHR platforms, only 22 use them to interact with medical professionals [9]. The implementation of such projects on a national or even international level requires considerable effort from professionals, governments, and the public. Implementation strategies must address key issues, including privacy and security, data standardization, algorithm sensitivity and specificity, as well as user training and ethical compliance. A study conducted in 2018 with the Organization for Economic Cooperation and Development (OECD) member countries as subjects showed an increase in the use of PHRs. In 2013, 13% of patients chose to make a medical appointment through the network; in 2024, the percentage increased to 43% in Europe [10,11]. Australia is a good example of a country that has initiated a PHR service where the system is connected to hospitals, physicians, pharmacists, medical analysis labs, and imaging centers. The growth in the number of users has been significant from 2018 to 2024, recording 16,920,000 patients. In terms of medical institutions, more than 90% of them use the service [12]. Another example is Sweden, which foresaw the potential of PHRs 20 years ago, and today, almost all medical institutions use PHRs [13,14]. These are just a few examples of good practice, but on the other hand, there are less developed countries whose digitization systems are underdeveloped. Turkey is also an example of good practice in PHR application management. A paper published in 2023 [15] presents an application that is easily accessible to the population. Most of the country’s healthcare institutions are connected to this application. Studies show that approximately 82% of the Turkish population uses this application to manage their health records. The application manages data from laboratory analysis, diagnosis, treatment, and imaging. It can even analyze specific symptoms that need to be monitored over time in chronic diseases. In addition, it can be connected to monitoring devices such as smartwatches to monitor particular parameters [15,16].

In contrast, Romanian EHRs are poorly utilized, although the implementation steps began in 2012, and the platform is currently used. Electronic health records (EHRs) are underutilized, despite implementation steps beginning in 2012. The platform is currently used by approximately 32,500 physicians and only 11,500 patients. A major problem is that most of the data stored in this system comes from general practitioners and not from specialists or medical institutions, which means that the data is not updated in real time [17].

The main contribution of this paper is the evaluation and review of different concepts and architectures underlying PHR systems, their benefits, challenges, adoption factors, and security and privacy concerns, taking into account technologies such as Internet of Things sensors, artificial intelligence, and blockchain.

This paper is organized as follows: first, there is a brief introduction related to the state of the art and the motivation of this study, followed by Section 2, where the main electronic health record systems’ current trends are presented. The benefits and challenges of personal health records are discussed in Section 3. In Section 4, the main data sources and system architectures are discussed and evaluated in detail. The Section 5 of the paper is represented by the conclusions, where the roadmap of PHR systems is discussed, and further improvements are proposed.

## 2. Electronic Health Record Systems: Current Trends

**Figure 1:**
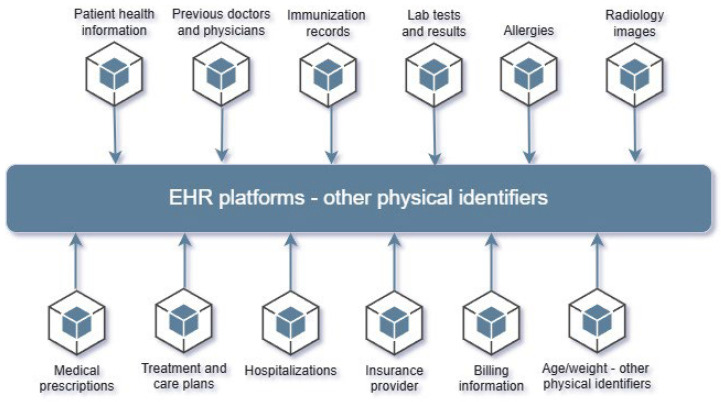
Main data points for an EHR platform.

The main force that keeps all the necessary data together is the Electronic Medical Record (EMR) [7]. In this sense, EMR or EHR (which also stands for electronic health record) is a way of storing the patient’s health data in an appropriate structure that allows for standardized manipulation in a computer system [2,18]. In a traditional way, the EHR has been defined as a digital version of a patient’s medical history that is maintained by healthcare providers over time [19]. In general, this means that the data recorded in hospitals or healthcare providers are based on the reported observations of the examination, diagnosis, and treatment during the patient’s visits (Figure 1). In other words, it represents a digital repository that keeps the patient’s health-related data as accurate as possible throughout his or her lifetime, accessible through the hospital’s informatics applications. In this respect, the patient’s health information is recorded, stored, and maintained by its creator (i.e., the medical staff or various hospital departments) [20]. This use case shows a unidirectional relationship between the patient and doctor, where the patient is completely dependent on the actions of the medical institution.

In hospital environments, the EHR is the most important tool for medical staff, keeping all patient information together, from imaging to laboratory data to blood analysis or data coming from multiparametric monitors, such as pulse, respiration rate, blood perfusion, lung ventilation [2], or any other medical information provided by wearable devices, including IoT sensors. The EHR is the main data collector that, through interoperability, links all clinical information system applications such as the PACS (Picture Archiving and Communication System), RIS (Radiology Information System), or LIS (Laboratory Information System), providing support to professional providers for patient management [21]. However, in recent years, the concept of EHR has evolved, capturing different perspectives defined by the International Organization for Standardization (ISO), especially at the level of architecture of services, systems, and communication of EHR [4]. An important aspect of all data is traceability, maintaining data quality through a functional and structural description to be queried in an interoperable way in different database structures [5,22].

Although the concepts of EMR (managed by a single healthcare provider), EHR (using multiple sources and sharing information between hospitals), and PHR (managed directly by the patient and including manually entered data) are distinct, they are often interconnected or combined in practice. Many modern PHR systems are connected to an institutional EHR, and some EHR platforms offer limited PHR functionality. In this context, conceptual distinctions remain useful, but actual implementations reflect a spectrum of integration between provider-controlled and patient-managed systems [23,24].

However, over the last two decades, there has been an increase in the number of patients who require access to their medical records as they travel or migrate across countries and continents, highlighting the limitations of current electronic health record systems. On the one hand, there are different institutional regulations regarding the privacy and security of accessed data from country to country. On the other hand, the internal structure of EHRs from healthcare providers is not consistent, and in this sense, it does not allow for data exchange in similar formats. This hinders interoperability among providers, which depends on the maturity of the local social health system to adopt and implement the standards [22,25]. The need for standardization is critical. Currently, disparate EHR platforms often operate in isolation, with different formats, data structures, and communication protocols, resulting in fragmented information exchange. This inconsistency not only hinders interoperability among healthcare providers but also creates challenges for patient safety, data integrity, and seamless care transitions. For healthcare providers, the inability to efficiently access and share patient data across systems can lead to delays, medical errors, and redundant testing, undermining the core goal of EHRs to improve care quality and outcomes.

The need for the standardization of EHR systems is therefore paramount. By establishing uniform standards and guidelines for data collection, storage, and exchange, healthcare organizations can ensure that EHR systems are interoperable, secure, and patient-centric. Standardized EHR systems would not only enable more accurate and consistent information exchange but would also foster better collaboration among healthcare professionals, improve patient safety, and provide better data for medical research and public health initiatives [26]. This article examines the urgent need for EHR standardization and explores its potential impact on healthcare efficiency, patient outcomes, and the patient–physician relationship. Moreover, it analyzes various architectures, concepts, and technologies that can increase efficiency and improve the security of PHR information. The requirements for these information systems were recently defined by ISO/IEEE 11073 [27] Personal Health Data Standard, EHR being referred to in this context as the Shared Health Record (SHR) or Health Information Exchange (HIE). Taking into account all the medical individual or interconnected systems that support the care of an individual, there is a lack of application of these standards [1].

The involvement of artificial intelligence (i.e., AI) in the management and processing of electronic health records is having a significant impact on what appears to be the future of medicine. The trend toward integrating AI into personal health records has many benefits for patient care modalities, including the ability to influence the diagnostic process. This not only enhances clinical decision making but also supports personalized treatment planning by analyzing individual patient profiles. EHRs typically generate large amounts of data, and AI helps make the information more efficient [27,28,29]. These large volumes of data are typically unstructured, derived from clinical observations, and their automatic processing with natural language processing (NLP) techniques results in the cleansing and structuring of the data [30]. Moreover, intelligent systems can extract clinically relevant information from physician notes, discharge summaries, and radiology reports—sources that have traditionally been difficult to process at scale. Additionally, predictive analytics enabled by AI can identify early warning signs and trends in disease progression, supporting preventive care strategies. In this matter, EHRs with AI are considered systems that can act as intelligent medical assistants, even in guiding diagnosis, treatment, and potential allergies. Some platforms are capable of offering real-time decision support, alerting clinicians about possible drug interactions, deviations from clinical guidelines, or emerging critical conditions. It can also predict a patient’s susceptibility to certain diseases based on their medical history. The efficiency of diagnostic methods is critical; it is now estimated that 1/3 of a clinician’s time with a patient is spent reviewing the medical record. However, the integration of AI involves significant risks, including the potential for algorithmic bias, a lack of transparency in decision making processes, insufficient clinical validation, and an overestimation of the accuracy of automated recommendations. Ethical concerns also arise, particularly regarding patient consent, data ownership, and the risk of reinforcing existing disparities in healthcare outcomes. Consequently, the integration of AI within PHRs must be accompanied by the implementation of comprehensive control frameworks, enhanced transparency measures, and rigorous validation processes to ensure the safety and fairness of its utilization [31].

It is clear that the integration of artificial intelligence into EHR/PHR systems contributes to more efficient patient care through better communication, improves productivity, and reduces medical staff burnout [32,33]. The use of AI will continue to develop and become more widespread. It is beneficial for managing large amounts of data. As progress is made, more specialists will be needed, and communication between patients and doctors will improve.

## 3. Personal Health Records Benefits and Challenges

One solution may be to involve the patient in the medical record management process, making them the owner of the record and giving them full access to its content. As a result, a patient-centric representation of the medical record would be appropriate, with authority over the content held by the individual. This brings into discussion the PHR [34,35]. The convergence of IoT, AI, and blockchain technologies is key to developing a smart PHR. IoT enables real-time health monitoring by continuously collecting data from medical and wearable devices (heart rate, blood sugar, and physical activity). Artificial intelligence enhances these systems by analyzing unstructured data, predicting disease progression, and providing personalized recommendations. Blockchain ensures that all data exchanges are secure. Together, these technologies support a patient-centric model of care [36,37,38].

The utilization of PHRs as a tool to digitize medicine on a large scale is a concept that shows great promise and has the potential to confer many benefits on the medical system. In practice, however, the real-world implementation of PHRs has proven to be slow, fragmented, and often unsuccessful. The complexity of implementation is not attributable to technical issues; instead, it is due to socio-behavioral, infrastructural, and organizational barriers [39]. As outlined in Table 1, several countries have attempted to implement national PHR systems, with varying degrees of success. While the My Health Record system in Australia boasts a coverage of over 90% [40] of the population, only a fraction of these records are actively populated with clinical data. A similar situation has been observed in countries such as Portugal [41] and the United Kingdom [42]. Early PHR initiatives have been poorly adopted due to a lack of promotion, usability issues, or minimal integration with existing EHR systems. A significant challenge arises from the behavioral opposition exhibited by patients and health professionals. Conversely, patients may lack the digital literacy skills, confidence, or motivation to utilize these tools effectively. In a considerable number of low- and middle-income countries, the digital infrastructure is inadequate for the large-scale deployment of such systems. Concerns regarding privacy, data ownership, and security further complicate this situation. Consequently, it is unsurprising that there are currently a few examples of fully functional and widely adopted PHR systems [43].

Historically, PHRs have evolved from EHRs due to the need for medical data personalization for patients who need continuity of care but in a non-hospitalized environment [35,45,46,47,48]. According to one definition, it has been stated that a PHR is a set of electronic or paper collections of records/data regarding an individual’s personal health status, from a variety of sources, and controlled or shared by that individual. Therefore, the medical information is captured by the individual and included in their record, giving them an active role in maintaining their health information [19]. Considering the definition of EHR, a first conclusion can be drawn that PHR is an extension of EHR, but in a more decentralized approach. Additionally, access to the clinical content can be shared by the individual with stakeholders (i.e., authorized physicians and private healthcare providers) to contribute to their care.

In a broader sense, PHRs function as electronic repositories or platforms for patients, where clinical information is collected and stored, enabling easy and standardized data communication between healthcare stakeholders [34,49,50]. A key reason for this is the continuity of care, with both medical and social content in the same place. This means that PHRs are not only collections of data provided by one or more EHR systems but also a place where medical devices and sensor wearables, mHealth technologies, telemedicine (i.e., teleconsultation or telediagnosis processes), and decision support systems are connected and integrated [51,52,53,54].

It is worth discussing the relationship between PHRs and a client or a patient as interchangeable entities, keeping in mind that a patient can be a client and vice versa at any given time. In Health Management Systems (HMS), the patient-PHR relationship can be seen as a new paradigm that empowers the patient to be in control of medical data, enabling a more secure self-decision process and ultimately improving healthcare outcomes. This comes with a set of rules regarding confidentiality and data security protocols, placing the client at the center of the HMS system. Therefore, it has been stated that the relationship between a patient and his PHR should have the main characteristics presented in the following section [55].

The patient is the owner of their past, present, and future PHR data, which ultimately gives them control over their health. Therefore, personal medical information, hereditary medical history, test/drug results, information contained in various electrophysiological signals delivered by diagnostic devices or wearables, along with the treatment information that builds the medical repository, are retained by the patient [48,56,57]. Because the data is proprietary, it can only be shared with specific permission policies.

Patients have the right to access and update the information in their PHR at any time. This can be achieved through electronic platforms, mobile applications, or in collaboration with medical professionals who can update records in real-time.

In this context, data confidentiality is a critical aspect of ensuring the privacy of medical data. The patient must be confident that their information is protected and that access to it is limited to authorized individuals, such as physicians and medical staff. Data collection and integration are also key aspects of a PHR. Medical information can come from multiple sources, such as medical records, laboratory test results, medical imaging reports, and even health monitoring devices (e.g., smart watches or health sensors) [35,58]. The PHR enables the collection and integration of this data in one place to provide a comprehensive radiography of the patient’s health. Additionally, the patient may choose to share specific information in the PHR with healthcare professionals, including primary care physicians, specialists, or other healthcare providers. This facilitates coordinated care, leading to better management of diseases or conditions. In this context, it is important to consider privacy and security issues. Recent studies have shown significant vulnerabilities to cyberattacks, accidental data loss, or unauthorized access. It is also meaningful to approach the development of PHR applications from this perspective, as it would increase patient confidence [59,60].

A PHR can include health monitoring capabilities, such as tracking vital signs, glucose levels, or blood pressure. This data can help patients better manage their health and make informed decisions. To accomplish this, the PHR must facilitate communication between the patient and healthcare professionals. For example, the patient can send questions or updates to their physician, who can respond and offer advice or adjust treatment plans.

In the event of a medical emergency, the PHR can contain vital information, such as allergies, prescribed medications, or pre-existing conditions, that can be critical in determining the best course of action. It can be quickly accessed by emergency personnel to provide appropriate care [19,58].

Sharing information with the patient can also be a pitfall to PHR adoption and sustainability. Medical information can sometimes be challenging to understand and may cause the individual to want to discontinue the use of the PHR. Therefore, the relationship between the type of patient (i.e., with a chronic disease, in an emergency, in need of home care, various types of disability, etc.) and the PHR should be much better understood. On the other hand, patients often do not understand the importance of the data and may not be analytical enough in the process of self-managing their health [6].

Several factors contribute to making a PHR sustainable over time. First, an adoption phase is critical [59]. The architecture of the PHR is essential to ensure that the patient will accept the interaction with the technological part [6,60]. In this way, some EMR elements need to be integrated into the PHR to enhance the patient’s personal experience. Second, it is important to make the use of the PHR interface as pleasant as possible. In this phase, the personalization of the PHR with respect to the patient’s approach to weighing things from a medical point of view will provide a degree of satisfaction to continue the self-management of health status.

## 4. PHR Data Sources and System Architectures

The type of data that should be included in the repositories is still a matter of debate. Considering that medical and patient wellness data come from a wide variety of sources, it is consistent, authentic, and truthful to be shared across different health care systems [54].

**Figure 2:**
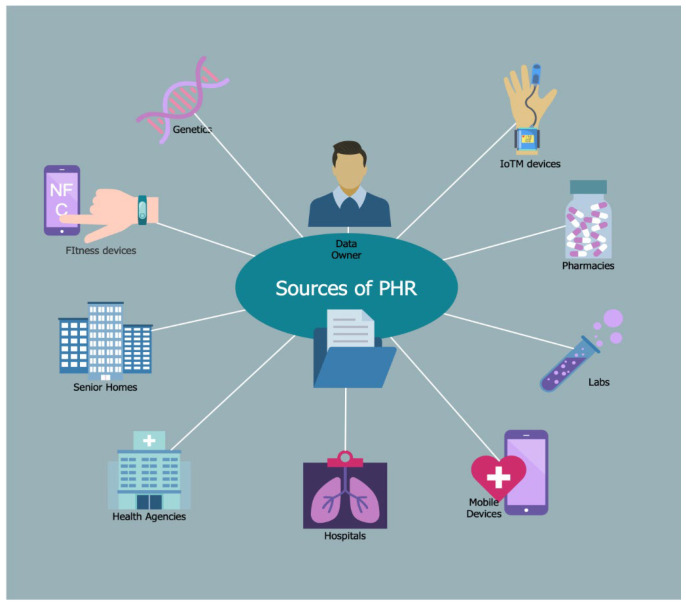
Personal health record data sources illustrating the variety of data sources that can be integrated.

Figure 2 presents the data sources that can be stored in PHR’s repositories and suggests a high level of integration of data types, ranging from personal devices to hospital EHRs.

Examples include information such as social status, family history, allergies, primary medical conditions, symptom lists, medications, laboratory tests, and any other data collected from wearable devices and healthcare Internet of Things (IoT) devices. We should mention that there are no data type limitations depending on the structure and meaning of the PHR [61,62,63,64,65,66,67,68,69]. Ideally, the content of the repositories should be populated with all relevant and valuable data for the patient to facilitate the processing of clinical information. An important aspect to consider is the origin of the data, which the patient or a clinical system can provide. In other words, source data types can be subjective or objective. For example, a simple manual entry of a blood pressure reading by the patient may result in an incorrect diagnosis. On the other hand, the same value automatically transmitted to the PHR can be trusted because it was recorded by a home or hospital medical device [58].

Therefore, in terms of data, the PHR can provide snapshots of a patient’s conditions or medications, depending on each individual’s specific needs. Vital signs (e.g., pulse, respiratory rate, blood pressure, and oxygen saturation), wellness information from IoT devices, lab or imaging data, or services from shared clinical records are integrated in a clinically appropriate manner.

There are a few accepted architectures for PHRs. For this purpose, untethered PHRs are represented by standalone or web/portal applications where the patient/client can log in to access their private medical records, which are usually stored on private servers. This is the case when private medical institutions or medical companies hold medical records. A simple example of a PHR is an application that can be installed as a standalone program on a computer, maintaining a database for imaging CDs or DVDs that a patient receives after a CT or MRI scan. Therefore, the patient can create his or her own PHR, with the main drawback that it cannot share information with other healthcare stakeholders (at least not easily, without advanced knowledge of PHR manipulation or programming).

A PHR can be considered tethered if different entities maintain the medical records [50]. Here, we can point to EMRs owned by hospitals through HIS (i.e., Hospital Information Systems), medical records owned by primary care physicians, or employers. On the other hand, PHRs need to exchange information, making interoperability a core functionality for this type of system. This means importing and exporting data in a standardized way (i.e., using the same communication systems and data standards). Interoperability standards for EMRs are based on ISO 13606 [70], HL7 v3 Clinical Document Architecture, HL7 v3 Care Provision Message for Record Exchange, the HL7 EHR System Functional Model ISO/HL7 10781:2009 [22], and the OpenEHR Foundation [71].

In the same manner, PHR design and quality guidelines must fulfill those standards. On the other hand, PHRs, being also electronic products, require software engineering, as IEEE 830-1998 [72] and ISO 9241-210:2019 [73], presenting recommendations for human-centered design principles, should be considered. At the same time, the FHIR (i.e., Fast Healthcare Interoperability Resources) standard introduces a simplified model for medical data, utilizing so-called resources as an important update to the data specifications [74]. Therefore, the exchange information infrastructure is based on 13 modules, such as Security & Privacy, Conformance, Administration, Clinical, Drugs, Diagnostics, Financial, Workflow, etc., with each module having several resources assigned to it. HL7FHIR exchanges information about resources in XML or JSON formats (or ND-JSON for large amounts of data) using the FHIR (Fast Healthcare Interoperability Resources) API.

With these standard requirements in mind, many enterprise healthcare EMRs, such as Epic or Cerner, have developed FHIR servers that integrate support for FHIR through HIE. In addition, the new SMART on FHIR (i.e., Substitutable Medical Apps, Reusable Technology) is creating healthcare ecosystems through simple web CRUD operations on the resources [75]. As a result, many applications have been developed that can interact and exchange clinical data with EMRs [76]. These typically include patient portals or telehealth platforms that provide an easy-to-use and seamless experience for patients. These are called ecosystem-based PHRs.

**Figure 3:**
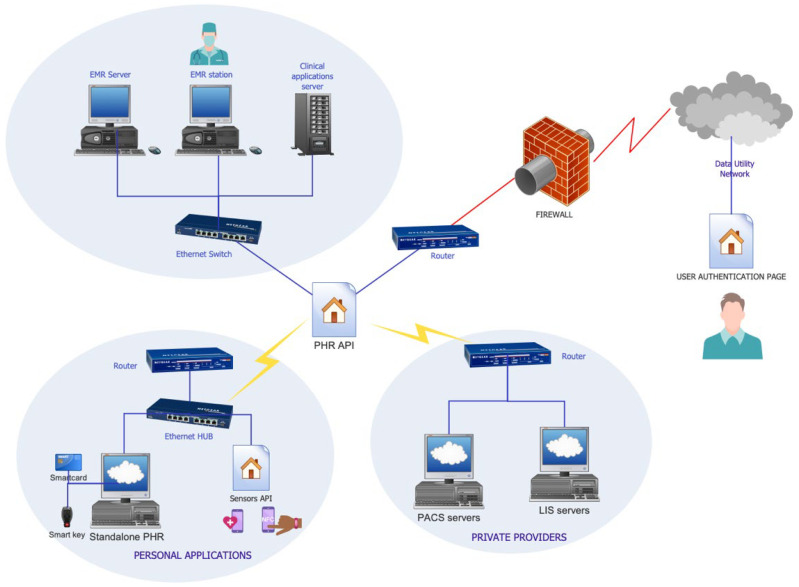
Architecture of a hybrid PHR system illustrating the integration and interoperability of medical information systems. The model emphasizes the role of the PHR as a communication interface between personal applications, private providers, and institutional clinical systems.

Figure 3 presents an architecture for an integrated, hybrid PHR system. Such a structure is collecting clinical data from multiple EMRs or from other health records, such as imaging providers or blood analysis labs, and wearable sensor data, allowing interoperability through an application programming interface API [77]. Other examples of interoperability include platforms that permit a PHR API to unify clinical and wearable API networks, such as Validic and Human APIs in the mHealth space [78,79]. This concept has broadened the path for mobile PHRs, which are usually apps that include many features, such as automatic blood pressure, pulse, respiration rate, appointment and medication reminders, and exercise tracking [80].

More recently, blockchain-based PHRs have been implemented, in which each record is stored as a block in a decentralized environment that ensures the privacy and security of clinical information. In this way, patients have control over access to their data [81]. There are also implementations of federated PHRs, where patient records are located in different places and can be accessed and integrated as needed. Blockchain architectures work with authorization models for medical assets, with smart contracts in the middle as an equivalent for the information exchange between patients and healthcare providers [60]. This is an approach for patients who may have medical visits in different institutions and private health care providers [82,83].

A revolutionary approach to managing and sharing healthcare data is the use of EHRs on a blockchain. This idea leverages the decentralized, secure, and immutable properties of blockchain to address some of healthcare’s biggest challenges, including data security, privacy, interoperability, and patient control over their health records.

Despite the considerable advantages that blockchain technology offers for PHRs, including immutability, decentralized access control, and traceability, its applicability must be subjected to rigorous scrutiny. In healthcare systems, authorized networks are utilized, with sensitive data stored off-chain and cryptographic references kept on-chain to ensure confidentiality. The challenges associated with interoperability between healthcare systems can be mitigated by leveraging blockchain capabilities. Furthermore, the issue of patient data control can be addressed by giving individuals greater autonomy over their personal information. However, traditional systems such as centralized electronic records, access logs, or data encryption offer adequate solutions in many situations. Consequently, the application of blockchain technology is not universally appropriate; its effectiveness depends on collaboration between multiple stakeholders (e.g., hospitals, clinics, and patients) who require a secure and immutable platform for inter-organizational interaction [84,85].

To enhance data privacy and security in decentralized PHR architectures, contemporary blockchain solutions are progressively depending on sophisticated cryptographic techniques. Zero-knowledge proofs (ZKPs) are a method of proving the possession of medical data without revealing its actual value, thereby enhancing privacy when verifying access rights [86]. Similarly, homomorphic encryption facilitates the processing of encrypted medical data without the need for decryption, thereby enabling secure analysis to be conducted directly on encrypted records [87]. In practical implementations, blockchain frameworks such as Hyperledger Fabric (a permissioned blockchain platform) and Ethereum-based solutions are utilized for their modular structure. For instance, in the OmniPHR model, a Hyperledger-based multi-chain approach was utilized to securely distribute encrypted patient records between healthcare nodes with minimal latency and without compromising confidentiality [82]. The immutability and decentralization of blockchain are key strengths, and the combination with applied cryptographic tools and standardized frameworks renders it truly viable for sensitive medical contexts.

**Figure 4:**
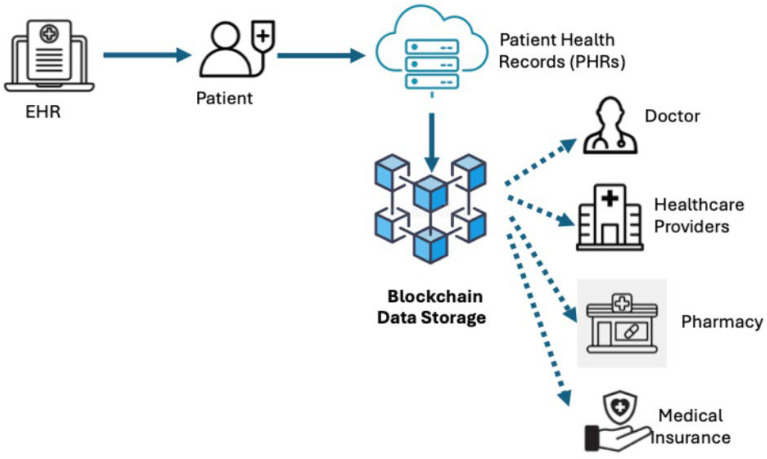
Structure of a blockchain PHR.

EHRs on blockchain leverage decentralization, security, and immutability to improve healthcare data management. Key features include decentralized data storage, which ensures that no single entity controls all records; enhanced security and privacy through encryption and immutable records; and interoperability, which enables seamless data sharing among healthcare providers. Patients gain control over their health data by deciding who can access it, while smart contracts automate specific processes, such as data sharing, based on patient consent. The goal of these features is to improve healthcare efficiency, data security, and patient empowerment. Figure 4 presents the architecture of a PHR based on advanced blockchain technologies.

### 4.1. Health Record Applications

According to the scientific literature, a significant reason for the slow adoption of PHRs worldwide is the closed nature of the systems [88,89]. We are not only talking about closed-source applications but also closed ecosystems. Proprietary file formats, proprietary communication protocols, and narrow standards for different PHRs have created a closed market that is not conducive to the widespread adoption of these technologies. This approach will limit the usefulness of the platform, its reach to the general population, and its adoption in larger global zones because the platform is often tied to a vendor ecosystem of support and service that is not always available or expensive enough to reach penetration in poorer regions. These aspects will have a negative impact on the privacy of individuals, as they are often seen only as a medical revenue factor. Data can be commercialized in various ways by private entities. The lack of openness in this industry is a major factor limiting the adoption of PHRs.

One interesting approach is the PHR ecosystem architecture. In this scenario, the company provides a platform that ensures the consistency and security of medical data through its middleware and API, but allows vetted third parties to provide functionality through various applications. One such example is Microsoft’s HealthVault system [89]. The system was able to store, manage, and share their health information in a secure, centralized system. The platform was designed to empower patients to take control of their health information, which could then be shared with healthcare providers, caregivers, and family members as needed. HealthVault is designed to store a wide variety of data, including medical records, prescriptions, fitness data, other data provided by wearable devices, allergies, and immunizations.

PHR platforms are either open-source or proprietary. In closed platforms or ecosystems, there are typically restrictions on the type of data that can be stored, the functionality of the applications, and the workflow. However, there are also no technical guarantees regarding privacy or the system’s resiliency.

From this perspective, an open architecture and ecosystem are the way to go. For example, in [89], an open PHR architecture is proposed that provides the fundamentals of medical data manipulation (transport, packaging, encryption, etc.) and enables easy extensibility of functionality through modules in an open manner. Another implementation is MyPHRMachines, which can be used in a decentralized, open way, and can manipulate medical data from DICOM (Digital Imaging and Communications in Medicine) files to genomic data and can use various services to interface with PHR terminal applications (the endpoint for the private patient). This application is designed to store lifelong personal health records in a secure fashion and allows access to third-party analyzing systems.

This architecture utilizes virtual machines to secure patient data and communicates with a generic hypervisor to start, stop, and clone virtual machines, as well as control their input and output by managing network access. These clones host our client’s PHR terminal application that interfaces with their data in a secure cloud topology (private or public, encrypted). This approach limits access to data to authorized agents and locations and limits misuse of the data by design.

Another similar platform for PHRs is Individo (a collaboration between Harvard Medical School and the Massachusetts Institute of Technology), but the system is considered more of a prototype than a working implementation. This platform is a subset of PHR systems called Personally Controlled Health Records (PCHRs), which allow patients to own a secure copy of their health records. Patients can also manage copies of their medical records as the custodian of the information. Access control is exercised by the user, who determines who has access to the information and who can modify or annotate it.

**Figure 5:**
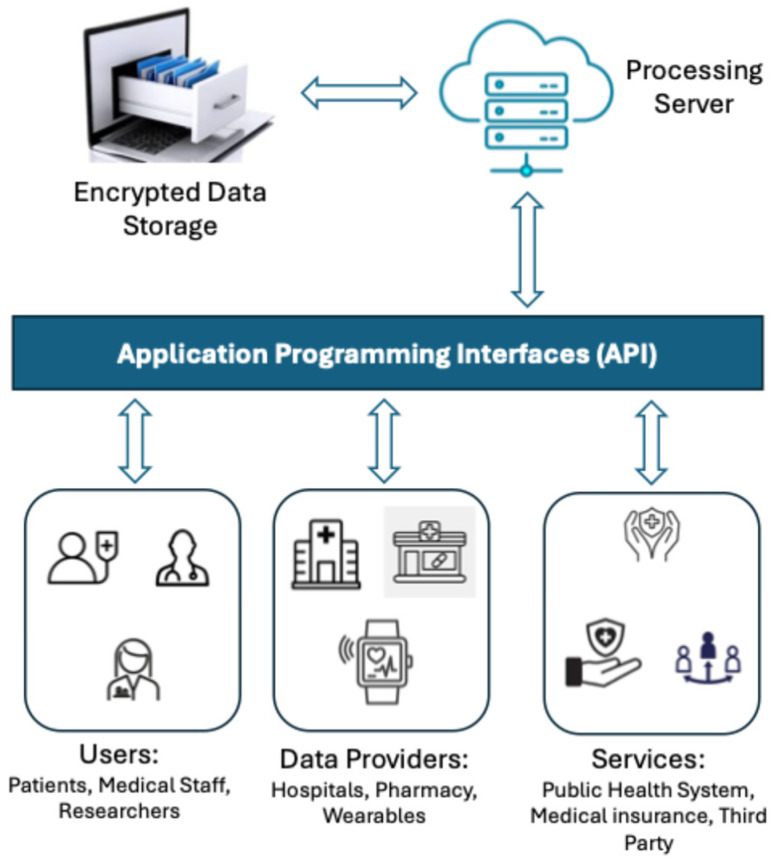
Personally controlled health record architecture.

Figure 5 shows the architecture, which includes the main encrypted data repository, the server, which is accessed by three entities through APIs interfaces: PCHR applications that serve patients, researchers, or healthcare providers; subscription agents that manage and deliver the data (hospitals, pharmacies, and other data providers); and service providers (public health systems, health insurance companies, and other third parties) [90].

**Figure 6:**
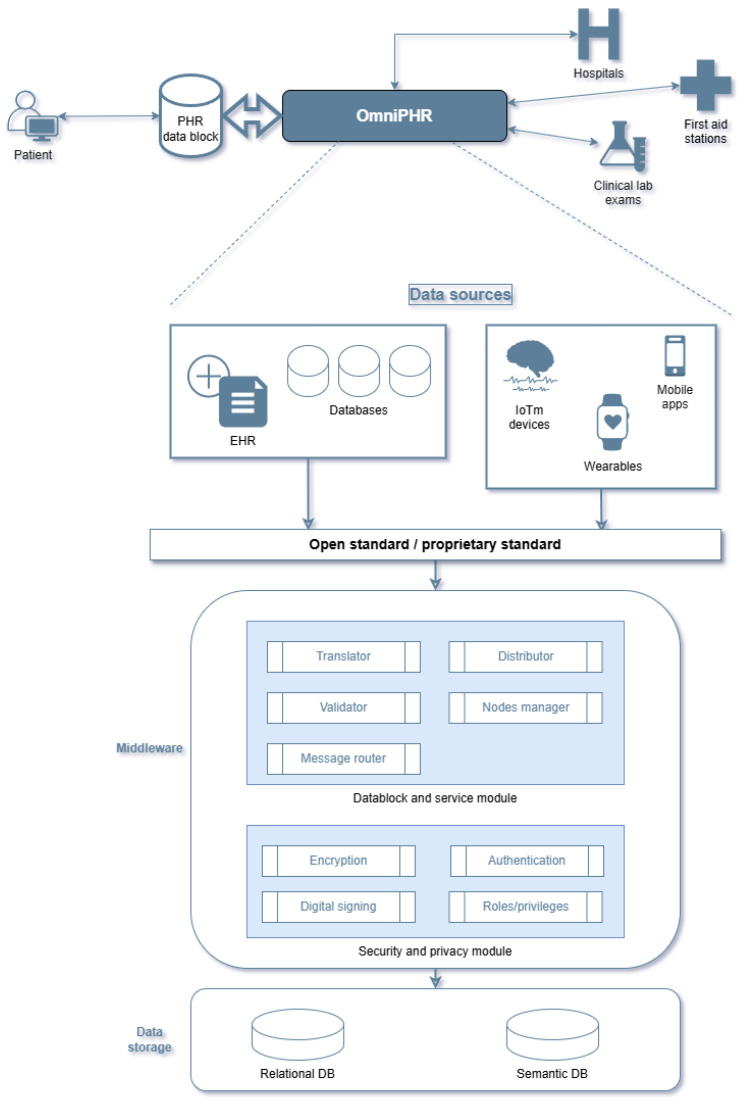
The architecture of the OmniPHR system, including the middleware, data sources, data storage, and key entities involved in accessing the information.

A more classical approach to this type of platform is described in [77]. In this case, the OmniPHR [91] model addresses the challenge of patients having health records scattered across multiple healthcare organizations, resulting in fragmented patient data and potential gaps in care. The model also addresses the challenge of healthcare providers who lack access to comprehensive and up-to-date patient information. In addition, the model aims to provide a single, unified view that can be accessed by both patients and providers, regardless of the technology or vendor used to store the data. The proposed model is designed to provide a distributed architecture for the secure, privacy-preserving, and interoperable exchange of personal health records and to mitigate problems and barriers to the adoption of PHR systems. The result is a more conventional approach than the previous one, which is described in Figure 6.

The OmniPHR model prioritizes the security of all stored data through the use of encryption and the careful management of administrative rights, allowing only authorized individuals to access data via a middleware layer (utilizing an API). The model can also recognize different formats and types of data used in the medical field. Legacy compatibility is a key feature of this model and can be implemented modularly at the middleware level. The architectures presented so far used an approach based on a middleware layer that acts as an intermediary between the low-level functionalities of the platform (regarding data manipulation and processing) and the higher-level tools that work directly with the individual. This approach appears to be optimal because it provides separation between the data and the applications used to input or output that data, thereby creating a layer of separation. Another type of architecture, as described in the literature, is based on microservices [75]. In this case, the platform (TreC) is internally monolithic but can provide a set of functionalities directly to the user through a dedicated set of services. In this case, there is a stronger coupling between the data and the processing layer, which can affect the scalability and modularity of the platform. However, the platform, although successfully implemented on a limited scale in the Province of Trento, is quite limited and implements its standards for data storage rather than using open standards and protocols already in use. This aspect limits the interoperability of the developed architecture.

All of these aspects can be improved with this well-designed security scheme for data storage and manipulation. Over the last decade, blockchain technology has matured and begun to be implemented across various industries. Other blockchain-based models have also been proposed for PHRs. For example, ref. [76] proposes a method for accessing personal health records in a secure and privacy-preserving manner. The method is based on ciphertext-policy attribute-based encryption (CP-ABE) and uses blockchain technology for accountability. The proposed method stores generated transactions in the blockchain to ensure data confidentiality and integrity. The healthcare provider verifies the integrity of the message by checking the blockchain. In addition, the scheme revokes users through a binary tree to resist collusion attacks. The cloud server updates the ciphertext according to the revocation list, thereby avoiding collusion between users and nodes while ensuring both forward and backward security. Therefore, using blockchain technology prevents data tampering and tracks malicious behavior, risks that are not well managed in traditional models.

Another proposal [67], implemented in a real system, enables the OmniPHR platform to ensure the distribution of patient data, supported by a blockchain network that enforces data immutability and authenticates the information transferred between different modules. Since the data can be tampered with in transit or when intercepted by malicious nodes, ensuring that the medical data is encrypted and guaranteed to originate only from authorized entities is a vital component of the network. The proposed multi-blockchain model, developed on top of the Hyperledger framework, can be used efficiently, with minimal delay—a recurring problem in blockchain transactions that involves different cryptographic and hashing algorithms across multiple nodes to ensure the authenticity of the given message—and without errors. Of course, the privileged information (patient data) must be encrypted to ensure sufficient privacy, given that blockchain hashes are public.

Another distributed application for health records management is My HealtheVet [92]. This web portal is designed to help US veterans navigate healthcare and related information. The architecture is modular, with the main functionalities being health management and data sharing. The portal allows for tracking and accessing medical prescriptions, interacting with physicians and doctors via an internal messaging system and appointment scheduler, and the aggregation of health records from various sources—internal doctors, external healthcare facilities, self-reporting systems, and more. A more modern system, based on a novel architecture, is the Patient Data Chain [93]. Its infrastructure is decentralized, incorporating blockchain technology to ensure a trust layer in the healthcare chain. The system links multiple healthcare providers and sources of data (personal medical records, laboratory test results, wearable devices, etc.), allowing aggregation into a single PHR platform where the user retains full ownership and control of the platform.

The system is built around Modex BCDB blockchain technology as middleware. Its purpose is to mediate access to the database layer that stores the data. The middleware works with the main app of the system—Patient Health Wallet—that allows storage and retrieval of data from the database. This app is considered the single source of truth in the system, being used to allow access to other systems that interact with the app—appointment schedulers, prescription storage/retrieval, etc. The patient allows access individually (via the app) for each access attempt and effectively manages data access, ensuring that privacy is respected. The external information is converted into HL7-compliant data, fused and stored internally, then retrieved and offered only for a limited scope when a request is made (each user of data only obtains access to what is deemed necessary, on demand). The system was validated in a closed clinical setting and allows enhanced data integration and interoperability with a limited number of EHR systems but is flexible and private by design. This, in turn, allows for further development and potentially its launch on the Romanian market.

### 4.2. Standards for Personal Health Records

There are many standards in use for implementing PHRs, most of which are derived from the EHR systems used in different regions [67]. Given the rigorous nature of the medical standards currently in use, it is preferable to move toward standards accepted by ANSI (American National Standards Institute) or ISO (International Standardization Organization)—which has established a committee for health informatics and publishes the ISO TC125 family of standards. In addition, HL7 International is an international standards development organization that produces the most widely used standards for healthcare interoperability—standards for the exchange, management, processing, and integration of electronic health information primarily for clinical and administrative purposes (typically used in EHR systems). For example, HL7 v2 is the most widely used healthcare exchange format for communicating data internationally in most hospitals that implement EHR systems. Version 3 (also known as v3/RIM) is the current version in use and is the most advanced and efficient.

**Figure 7:**
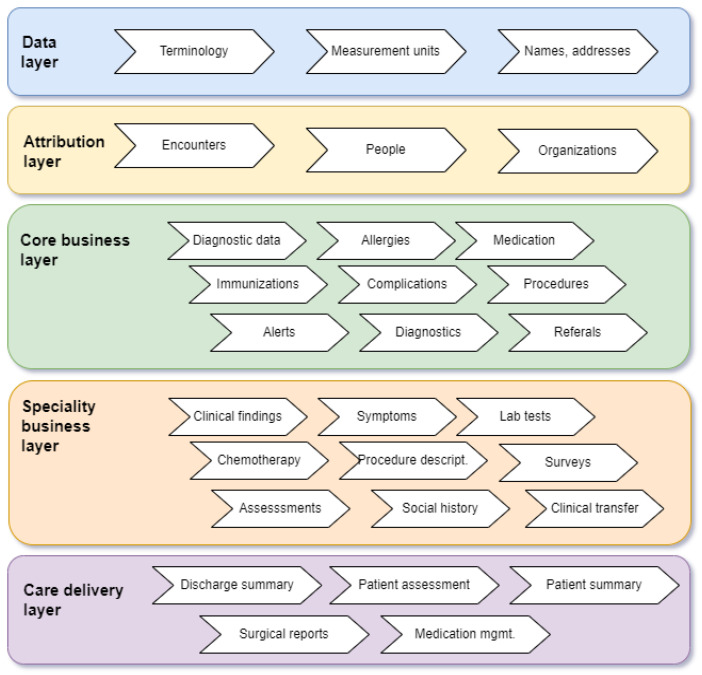
Architecture diagram for FHIR healthcare resources.

Typically, the exchange messages are used in conjunction with service-providing protocols that allow one software product to interface with another by exchanging messages. FHIR (Fast Healthcare Interoperability Resources) is one such protocol used to interface with cloud-based systems through RESTful interaction. FHIR provides a set of resources (for conformance, infrastructure definition, healthcare resource management, clinical, and financial) and an architecture for healthcare information management (Figure 7) [94].

There are also technology-agnostic architectures proposed by international nonprofit organizations, standardized by ISA and ANSI, such as OpenEHR [95], that can be considered when building health management platforms. Using such implementations increases the chances of interoperability between a given platform under development and other platforms/models already in use.

An important aspect to consider is the use of an integration engine. The role of an integration engine is primarily to convert messages exchanged between different EHR/PHR systems from one format to another. Its main goal is to ensure interoperability of the systems. There are many solutions to this problem, the most widely used being NextGen’s Mirth Connect, which offers an open and free implementation [96]. Other implementations include interface Ware’s Iguana (commercial products) [97]. An integration engine typically performs more functions: converting medical data formats, converting exchange messages, routing data to appropriate people, and writing to and reading from databases. Because a PHR works with different types of data and interfaces with EHRs or PHRs, implementing an integration engine is very important to the architecture of the platform being developed. A definite advantage is the simplification of the code base and development time, since the interconnection component is already developed.

From 2020 to 2024, significant efforts were made at the public policy level to achieve PHR interoperability. In the US, in addition to implementing the USCDI (US Core Data for Interoperability) [98], which defines the essential types of information that must be shared interoperably, the categories of essential data were expanded (USCDI versions 2 and 3 added data such as social determinants, detailed immunizations, genetic data, etc.). In the European Union, significant efforts have been made to establish the European Health Data Space (EHDS) [99,100], a legal and technical framework inaugurated in 2022 that emphasizes establishing common rules, infrastructures, and standardized protocols at the European level. The proposed EHDS regulation underscores the necessity to adopt international interoperability standards, explicitly referencing the HL7 FHIR, openEHR, and OMOP formats for research data [101,102]. A series of pilot projects [103,104] have been initiated (X-eHealth, Hospitals on FHIR, etc.) to facilitate compliance with these standards among Member States. In addition, eHAction has formulated a strategy recommending the use of unified terminologies (SNOMED CT, EDQM, etc.) and minimum clinical data sets (e.g., European Patient Summary based on IPS). Also at the international level, SNOMED International has entered into agreements with HL7 and openEHR to facilitate the use of SNOMED terminology in FHIR profiles and openEHR archetypes, removing licensing and technical barriers [105,106]. These developments indicate an unprecedented acceleration of standardization in recent years, with direct benefits for PHR interoperability [107].

IT systems used in healthcare must communicate efficiently, comply with confidentiality requirements, and be adapted to different cultural and technological contexts. To achieve these objectives, standards have been developed covering areas such as clinical data exchange, semantic information modeling, data security and confidentiality, medical device integration, and the internationalization of user interfaces [108]. Table 2 provides a summary classification of the main international standards used in e-health [108].

Standards such as HL7, FHIR, or ISO 13606 form the technical foundation for the interoperability of personal electronic health records. Comparative analysis shows the importance of choosing the right mix of standards depending on the implementation context: a national PHR system may require the robustness of archetype-based models, while an individual PHR application can be integrated using FHIR. Interoperability achieved through robust standards will enable the creation of a portable and comprehensive PHR that the patient controls but is also useful to clinicians on a global scale, with benefits for both patients and the entire healthcare system [123].

## 5. Discussion and Conclusions

One of the most problematic observations about PHRs in the scientific literature is that the main obstacles to increasing their use are the lack of openness in the ecosystems created to date and the absence of consistent standards for data storage and processing. In this regard, the creation of a platform that uses standards in the medical domain—open for use, modification, and extension—is critical for adoption. The interoperability of the various systems used in the medical (and related) fields, as well as the storage formats for processed data, are currently open issues. The systems produced by companies tend to be closed systems that lock users’ data into limited ecosystems. Because many hospitals (or medical networks) are not as well funded and do not use vertically integrated systems produced by the same company due to cost constraints, the penetration of complex PHR systems lags behind the overall trends of global digitization.

Standardization efforts (such as HL7 FHIR) can enable industry-wide collaboration on a larger scale, enabling communication between healthcare providers and patients through different implementations that can communicate easily and at a lower cost [124].

It was also noted that security needs to be built into the platform from the beginning. Given the legal restrictions on medical data processing, security cannot be seen as an add-on to be implemented later. There are few alternatives, and the latest technologies in security research (e.g., blockchain) are often missing from proposed platforms or models, despite being a viable option in terms of authentication and data immutability. Building trust with users (especially today, when personal information is a highly sought-after commodity for data processing companies) requires robust security measures and transparent data ownership policies [125].

In addition, considering the traditional approaches to implementing PHRs, namely, web applications, desktop applications, smartphone applications, or cloud-based platforms, each with its strengths and weaknesses, some recommendations emerge. For maximum impact from a technology perspective, a cloud-based platform is the best option for capturing, encrypting, storing, and processing the medical data for PHRs.

A smartphone ecosystem based on IoT devices is best used to interface with the end user, the patient (since the smartphone is now a basic tool available to almost everyone) [126]. PHRs are most effective when patients are actively involved. However, digital literacy and comfort levels with technology vary. Some users, including patients and healthcare professionals, may find entering data and managing their PHR to be inconvenient. Usability must be a priority to ensure widespread adoption.

In addition, social inequalities are an issue that should be addressed. Not everyone has equal access to technology or the digital literacy to manage a PHR. This could exacerbate existing health disparities. Efforts must be made to ensure inclusive design and to bridge the digital divide for all potential PHR users. This should be a priority, especially in the post-pandemic world; ignoring large populations in areas that may be epidemiologically active is not a good approach in a global society.

The lack of an infrastructure for HMS interoperability has shown that it is impossible to make rapid decisions about the health status of the population in the face of a pandemic situation [74]. In this regard, PHRs in correlation with EMRs integrated at a significant level could give an appropriate answer for individual health [127]. Therefore, it is necessary to explore the possibility of personalizing a set of medical information repositories for such situations. In addition, it is important to study in depth the interaction between patients and caregivers through the PHRs.

Another solution would be to adopt an innovation model using a PHR tool to assess the patient–physician relationship [2]. Patient empowerment can improve the above patient–physician paradigm. More recently, the assessment of the role of privacy, security, and usability has been discussed using an acceptance technology model [128,129]. These studies support the notion that the perceived ease of use has a direct impact on the use of PHRs. As a result, PHRs have emerged to improve communication and collaboration between physicians and patients. However, there are common principles that must be followed to ensure the success of this approach. Common principles for effective physician–patient interaction using PHRs include transparency and openness in communication, collaboration and mutual involvement, accessibility and ease-of-use platforms, and respect for patients’ right to confidentiality [130]. In addition, patients should be informed about how their medical information will be used and shared in the system of records. Their consent must be voluntary and informed. With this in mind, patients should have control over their medical information and be able to decide on its access and disclosure [6,73]. They should be able to choose who has access to their information and with whom to share it. This largely depends on the implementation of interoperability standards to ensure that different medical systems can communicate and share data in a coherent manner.

However, challenges arise when the privacy and security of clinical information are not assured. Maintaining the confidentiality of personal medical information is essential. Health records must be protected from unauthorized access and potential cybersecurity breaches. At the same time, it is well known that many healthcare systems store medical information in various formats and systems. Ensuring adequate data connectivity and interoperability is a complex technical challenge. The adoption of technological change is also a significant challenge. Not all patients and physicians are familiar with and comfortable using electronic records. It is important to facilitate technical adoption and ensure that implemented PHRs are easy to use and accessible [59].

The primary beneficiaries of PHR systems are the patients themselves. There are several benefits that patients can derive from using such electronic systems. One aspect is improved health management, as patients can easily access and track their medical history, medications, allergies, and lab results, empowering them to be more involved in the management of their health conditions and thus improving their health outcomes (and potential future personal and societal costs) in the long term [131].

Another benefit relates to informed decision making, given the abundance and accessibility of medical information. Patients can ask better questions, understand treatment options more clearly, and participate in shared decision making with their physicians, making them more likely to complete the entire course of treatment. This also allows for a more trusting relationship between the patient and physician, given the significant social problems posed by modern disinformation trends.

PHRs can improve patient–provider communication. Patients can use PHRs to share relevant information before appointments, track progress after visits, and clarify questions or concerns. This can reduce time and unnecessary appointments, increasing efficiency and lowering costs. PHRs ensure the continuity of care when patients move between healthcare providers. PHRs ensure that their medical history travels with them. This promotes smoother care transitions and avoids the unnecessary repetition of tests or procedures, thereby reducing costs and providing a more comprehensive view of a patient’s overall health.

Despite the progress made thus far, both in terms of technological advancement and the implementation of e-health records in various regions, there are still several gaps in the implementation and management of such records. There is a clear need for studies assessing the real impact of PHRs on health outcomes and healthcare costs. Furthermore, it is recommended that subsequent research efforts focus on exploring standardized evaluation frameworks for usability and accessibility, with a particular emphasis on vulnerable populations. Further investigation is required into a secure and decentralized architecture. Such an investigation should focus not only on technical feasibility but also on governance models, consent management, and user trust. A summary of the recommended future approaches for PHR is highlighted in Table 3.

In conclusion, PHR systems represent an essential step toward digitized, personalized, and patient-centered medicine. The integration of technologies such as artificial intelligence and the Internet of Things offers significant opportunities for healthcare. However, the widespread implementation of these systems requires overcoming significant obstacles related to interoperability, security, standardization, and user adoption. Future research and development should aim to build open, scalable, and secure applications that support effective interaction between patients, professionals, and institutions, thereby delivering benefits in terms of clinical outcomes and health system efficiency.

## Figures and Tables

**Table 1:** PHR implementation in different countries: types of architecture, obstacles, and effective use.

Country	PHR System	Architecture Type	Challenges	Utilization Rate	Refs.
USA	Multiple (e.g., HealthVault, Dossia, PHR connected through patient portals)	Centralized (HealthVault), Open-source (Dossia-Indivo)	Reluctance from the medical profession, lack of interoperability between systems	Estimated adoption rate >75% in 2020	[43]
UK	ePHR integrated with online general practice services (prescriptions, data access)	Centralized (tethered ePHR-connected to NHS systems)	Data protection concerns	28% in 2019	[41,44]
Sweden	Journalen–full access to EHR via patient portal	Centralized (integrated into the national EHR infrastructure)	Reluctance on the part of medical professionals (concerns about patient anxiety and loss of control)	Over 3.7 million users (37.9% of the population) by 2017; usage on the rise	[13]
Portugal	National web-based PHR	Centralized (opt-in, partially connected with EHR)	Lack of promotion and poor integration with EHR	3 months after the official launch (May 2013), ~109,600 people, ~1% of the country’s population	[41]
Turkey	E-nabiz is a national PHR connected with all medical centres.	Centralized	Infrastructure development and integration of diverse systems	82% of the population actively using e-Nabız in 2023	[15]
Australia	My Health Record	Centralized	Privacy concerns citizens: engagement of providers and patients remains low despite high registration rates	22.8 million records created (~90% of the population) by 2020; only ~10 million contain clinical data	[40]

**Table 2:** Classification of international standards used in e-health and PHR.

Standard	Scope of Application	Objective
HL7 v2/v3 [109]	Standards for interoperability and data exchange	Exchange clinical data between information systems
FHIR [110]		Modern, RESTful API
ISO/HL7 27931:2009 [111]		Data exchange
DICOM [112]		Standard for medical imaging
EN ISO 21090:2011 [113]	Standards for data modeling and semantics	Standardized data types for information exchange
CEN/TR 15212:2006 [114]		Standardized vocabulary procedures
ISO/TR 20514:2005 [115]		EHR/PHR definition and context
ISO 27799:2008 [116]	Privacy and security standards	Health information security management
ISO/IEC 27002 [117]		General guide to information security
W3C P3P [118]		Privacy policies in web apps
ISO/TS 14265:2011 [119]		Classification of purposes of processing personal health data
ISO/IEC 25010 [120]	Standards for internationalization (i18n) and accessibility	Software quality, including internationalization
ISO 9241-151 [121]		Accessible and internationalized web interfaces
ISO/IEEE 11073 [27]	Standards for medical devices and IoT integration	Communications between medical devices and IT systems
IEC/TC 62 [122]		Electrical equipment used in medical practice

**Table 3:** Recommendations for improving the functionality, interoperability, and adoption of PHR systems.

Section	Recommendation
Design and functionalities	Improve the user interface Integrate predictive analytics features
Data quality	Standardize patient input to ensure consistency and validity (automatic validation and NLP for correction of patient-entered data) Consolidate data from multiple sources (devices, clinics, mobile apps)
Interoperability	Utilize standards (e.g., FHIR, HL7, and ISO 13606) SMART on FHIR for modular applications
Data protection	Implement advanced security measures (encryption, anonymization, access control)
Integration with AI and IoT	Use AI for analysis, but with rigorous validation Adapt PHR systems to integration with IoT devices for real-time monitoring
Institutional support	Actively involve service providers and insurance companies in promoting and supporting PHR
Validation	Assess the impact of PHR on clinical outcomes and healthcare costs Use multicenter randomized trials for clinical effectiveness
